# Genotype-guided warfarin dosing may benefit patients with mechanical aortic valve replacements: randomized controlled study

**DOI:** 10.1038/s41598-020-63985-7

**Published:** 2020-04-24

**Authors:** Kyung Eun Lee, Jeong Yee, Gwan Yung Lee, Jee Eun Chung, Jong Mi Seong, Byung Chul Chang, Hye Sun Gwak

**Affiliations:** 1 College of Pharmacy, Chungbuk National University, 660-1, Yeonje-ri, Osong-eup, Heungdeok-gu, Cheongju-si 28160 Korea; 2 College of Pharmacy and Graduate School of Pharmaceutical Sciences, Ewha Womans University, 52 Ewhayeodae-gil, Seodaemun-gu Seoul, 03760 Korea; 3 College of Pharmacy, Hanyang University, 55 Hanyangdeahak-ro, Sangnok-gu Ansan, 15588 Korea; 4 Department of Thoracic and Cardiovascular Surgery, Bundang CHA Medical Center, CHA University, 59, Yatap-ro, Bundang-gu Seongnam, 13496 Korea; 5 Department of Thoracic & Cardiovascular Surgery, Yonsei University Medical Center, 50-1 Yonsei-ro, Seodaemun-gu, Seoul, 03722 Korea

**Keywords:** Interventional cardiology, Genetics research

## Abstract

This prospective, single-blind, randomized study was designed to evaluate the effect of genotype-based warfarin dosing compared with standard warfarin dosing in Korean patients with mechanical cardiac valves. Patients were assigned to either the genotype-based dosing group or the standard dosing group using stratified block randomization. The genotype-based dosing equation was adopted from a previous study which included VKORC1 rs9934438, CYP2C9 rs1057910, CYP4F2 rs2108622, and age. Primary outcomes included the percentage of time in the therapeutic range (pTTR): (i) during the first week following initiation of warfarin therapy, (ii) during hospitalization and (iii) until the first outpatient visit. A total of 91 patients were included in the analysis, 42 treated with genotype-based warfarin dosing and 49 treated with standard warfarin dosing. The genotype frequency differences of the three SNPs included in this study (ie, VKORC1, CYP2C9, CYP4F2), between the genotype-based dosing and standard dosing groups were not different. The genotype-based dosing group trended toward higher pTTR when compared with the standard dosing group, although this difference was not statistically significant. In patients with aortic valve replacement, TTR_Traditional_ and TTR_Rosendaal_ were significantly higher in the genotype-based dosing group when compared with the standard dosing group during the first week following treatment initiation [ie, 58.5% vs. 38.1% (p = 0.009) and 64.0% vs. 44.6% (p = 0.012), respectively]. Based on the results, the genotype-guided dosing did not offer a significant clinical advantage, but a possible benefit in patients with aortic valve replacement has been suggested.

## Introduction

Thrombotic and thromboembolic complications remain important causes of morbidity and mortality following mechanical valve replacement surgery, with a first-year incidence of 24%, and an incidence between years two and four of 15%, decreasing thereafter^1,2^. Thrombi can be detected by transesophageal echocardiography as early as nine days after mechanical valve replacement; morbidity and thromboembolic complications are more likely for thrombi occurring early^3^. In a study including patients with mechanical aortic valve replacement, 3% presented with transient ischemic attacks, permanent strokes, and peripheral events before discharge^4^. To avoid thromboembolic complications, the initiation of warfarin therapy after mechanical cardiac valve replacement surgery is a long-established standard of care with strong data to support its use, even with the advent of newer anticoagulation drugs^5,6^. However, warfarin has a narrow therapeutic index and inappropriate dosing significantly increases the risk for thromboembolism, bleeding, and hospitalization, particularly during the initial period of warfarin treatment^7,8^.

Warfarin dosing algorithms have led to improvements in predicting appropriate doses for patients. The incorporation of genetic variants into the clinical algorithm predicting optimal warfarin dosing has further improved predictability of appropriate doing and has been included in warfarin labels in various countries^9^. Furthermore, prospective trials comparing genotype-guided dosing with standard dosing were conducted to evaluate the significance of pharmacogenetic testing of warfarin. The vast majority of literature on genotype-guided warfarin dosing is in populations of European ancestry^10^. However, algorithms that do not include variants important to other group (eg, Asians) are unlikely to benefit these patient populations.

Moreover, various factors can influence the effectiveness of genotype-based dosing, and in patients with mechanical cardiac valve replacement, surgery-related factors may play a role. Valve position can affect patient prognosis after surgery since there are differences in the function of each valve, yet a limited number of studies are available which focus on the significance of the valve position. Therefore, in this study, we adopted genotype-guided dosing based on a previous study performed in Korean patients and aimed to evaluate the clinical utility of a genotype dosing algorithm compared with standard warfarin dosing in patients with mechanical cardiac valve replacements.

## Methods

### Study design

This prospective, single-blind, randomized study was designed to compare pharmacogenetic-guided dosing and standard dosing in patients being initiated on warfarin. From January 2013 to July 2017, patients age 18 years or older who were scheduled to undergo heart valve replacement surgery and planned to receive anticoagulation therapy with warfarin at Severance Cardiovascular Hospital of Yonsei University College of Medicine were screened for enrollment. Exclusion criteria were history of liver or kidney diseases, malignancy or warfarin use within 3 months of enrollment in the study.

Patients were assigned to either the genotype-based dosing group or the standard dosing group using stratified block randomization. Strata were defined as a combination of sex (male vs. female) and age (≥65 vs. <65 years); the block size was 6 subjects and patients were blinded to their assigned arm. Informed consent was obtained from all participants prior to the enrollment. The study would be terminated early if there were serious safety issues. The study was reviewed and approved by the Ethics Committee of the Severance Hospital Institutional Review Board and conducted in accordance with the Declaration of Helsinki and Good Clinical Practice guidelines (IRB 4-2012-0612). The study was registered on the CRIS (Clinical Research Information Service, http://cris.nih.go.kr, ref: KCT0004586) on 30/12/2019.

Genotyping for CYP2C9 (rs1057910), VKORC1 (rs9934438), and CYP4F2 (rs2108622) was performed using peripheral blood samples collected prior to the heart valve replacement surgery. Patients were prescribed heparin and bridged to warfarin one day after the surgery. The initial warfarin dose for the standard dosing arm was 5 mg, and personalized dosing for patients in the genotype-based arm were calculated using a regression equation developed in our previous observational study^11^. The model yielded the following equation: estimated initial warfarin dose (mg) = 11.305–2.082 × (number of VKORC1 rs9934438 T allele) − 1.615 × (number of CYP2C9 rs1057910 C allele) − 0.037 × age (year) + 0.983 × (CYP4F2 rs2108622 AA = 1, GA or GG = 0). International normalized ratio (INR) readings were checked on a daily basis during hospitalization and subsequently according to treating physician preferences.

### Genotyping

Genomic DNA was isolated from EDTA-blood samples using the QIAamp DNA Blood Mini Kit (QIAGEN GmbH, Hilden, Germany) according to the manufacturer’s protocol. The TaqMan genotyping assay was conducted using the real time PCR system (ABI 7300, ABI, Forster City, CA, USA) according to the manufacturer’s protocol.

### Endpoints

The primary outcomes of the study were the percentage of time in the therapeutic range (pTTR): (i) during the first week after initiation of warfarin therapy, (ii) during hospitalization and (iii) until the first outpatient visit. The pTTR was calculated using two methods, the traditional method (pTTR_Traditional_) and the Rosendaal method (pTTR_Rosendaal_). pTTR_Traditiona_ is a ratio defined as the number of INR values in the therapeutic range divided by the total number of INR measurements. pTTR_Rosendaal_ is defined as the number of days within the therapeutic range divided by the total number of days in the observation, using a standard linear interpolation method between successive INR values^12^.

The target therapeutic INR range was 2.0 to 3.0. To avoid overcorrections and in consideration of measurement error, INR readings smaller than 1.8 or greater than 3.2 were considered out of the therapeutic range^13^. The number of INR values in the sub-therapeutic range (INR < 1.8) and supra-therapeutic range (INR > 3.2) were retrospectively analyzed to identify any potential trends relating to the out-of-range INR values.

### Statistical analysis

Comparisons between two groups were made using the independent samples t-test (continuous data), and the chi-squared or Fisher’s exact tests (categorical data). Additional analysis was conducted to analyze the effect of genotype-based dosing in patients with different valve position. Sample size was calculated to be 102 for having an 80% power of detecting difference in rate outside target INR of 25% between the two groups at the 5% level of significance^14,15^. We assumed a prevalence rate outside target INR of 40% in the group having the least successful target INR. A p-value of <0.05 was considered statistically significant. All analyses were performed with the IBM SPSS Statistics version 20 Software (International Business Machines Corp., New York, USA).

## Results

A total of 125 patients were screened; the final cohort consisted of 42 and 49 patients in the genotype-based dosing group and standard dosing group, respectively. Of the 34 who were excluded, 3 died after surgery, 3 did not undergo operation, 2 were not prescribed warfarin, 7 did not adhere to the study protocol, and 19 (11 in the genotype-based dosing group, and 8 in the standard dosing group) received a low dose of warfarin because of their clinical status after surgery (ie, low body weight and intensive care unit admission). The median patient age was 57 years (range, 21–84), and the majority of patients were male (65.9%). The most common site of valve replacement was mitral valve (47.3%) followed by aortic valve (37.4%). Hypertension was the most prevalent comorbidity among the study participants (48.4%), and most patients were prescribed diuretics (92.3%) (Table 1). With the exception of diabetes—which was more common in the standard dosing group (p = 0.028)—baseline characteristics were well-balanced between the study groups. No differences were observed in the genotype distributions of the three SNPs tested (ie, VKORC1, CYP2C9, CYP4F2) between the groups (Table 2).

**Table 1:** Baseline characteristics of study population.

	Genotype-based dosing group (n = 42)	Standard dosing group (n = 49)	P-value
Sex, n (%)			0.562
Male	29 (69.0)	31 (63.3)	
Female	13 (31.0)	18 (36.7)	
Age, years	54.6 ± 14.8	57.2 ± 16.7	0.436
Weight, kg	64.0 ± 10.8	67.3 ± 13.4	0.207
Body mass index, kg/m^2^	23.2 ± 3.3	24.5 ± 3.9	0.083
Valve position, n (%)			0.992
Aortic	16 (38.1)	18 (36.7)	
Mitral	20 (47.6)	23 (46.9)	
Double^a^	4 (9.5)	5 (10.2)	
Tricuspid^b^	2 (4.8)	3 (6.1)	
Valve type, n (%)			0.232
C-ring	9 (21.4)	8 (16.3)	
C-E ring	12 (28.6)	9 (18.4)	
Sorin	5 (11.9)	2 (4.1)	
St Jude Medical	7 (16.7)	12 (24.5)	
Others^c^	9 (21.4)	18 (36.7)	
Current smokers, n (%)	10 (23.8)	9 (18.4)	0.524
Current alcohol drinkers, n (%)	10 (23.8)	10 (20.4)	0.696
Comorbidity, n (%)			
Hypertension	20 (47.6)	24 (49.0)	0.897
Diabetes	2 (4.8)	10 (20.4)	0.028
Congestive heart failure	3 (7.1)	4 (8.2)	1.000
Atrial fibrillation	4 (9.5)	10 (20.4)	0.151
Myocardial infarction	0(0)	0(0)	—
Co-medication^d^, n (%)			
INR-increasing drugs^e^	3 (7.5)	1 (2.0)	0.322
INR-decreasing drugs^f^	0(0)	1 (2.0)	1.000
Antiplatelet drugs^g^	3 (7.1)	7 (14.3)	0.331
ACE inhibitors or ARBs	26 (65.0)	26 (53.1)	0.256
Calcium channel blockers	2 (4.8)	5 (10.2)	0.445
Diuretics	38 (90.5)	46 (93.9)	0.699
Beta-blockers	12 (30.0)	21 (42.9)	0.212
HMG CoA receptor inhibitor	7 (17.5)	15 (30.6)	0.154

^a^Aortic plus mitral valve.

^b^Any valve replacements including the tricuspid valve.

^c^Including Saddle, ATS, OnX, and prostheses using two or more different valve type.

^d^There were 2 missing data for co-medication.

^e^Including amiodarone, doxifluridine, and fluconazole.

^f^Including carbamazepine, phenytoin and rifampin.

^g^Including aspirin, cilostazol, and clopidogrel.

**Table 2:** Comparisons of genotype results of study patients between genotype-based dosing group and standard dosing group.

	Minor allele frequency	Genotype	Total	Genotype-guided dosing group	Standard dosing Group	P-value
*VKORC1*	0.08	CC	2 (2.2)	0(0)	2 (4.1)	0.370
rs9934438 (C > T)		CT	10 (11.0)	4 (9.5)	6 (12.2)	
		TT	79 (86.8)	38 (90.5)	41 (83.7)	
*CYP2C9*	0.03	AA	85 (93.4)	40 (95.2)	45 (91.8)	0.683
rs1057910 (A > C)		AC	6 (6.6)	2 (4.8)	4 (8.2)	
*CYP4F2*	0.31	GG	43 (47.3)	21 (50.0)	22 (44.9)	0.698
rs2108622 (G > A)		GA	39 (42.9)	18 (42.9)	21 (42.9)	
		AA	9 (9.9)	3 (7.1)	6 (12.2)	

**Figure 1:**
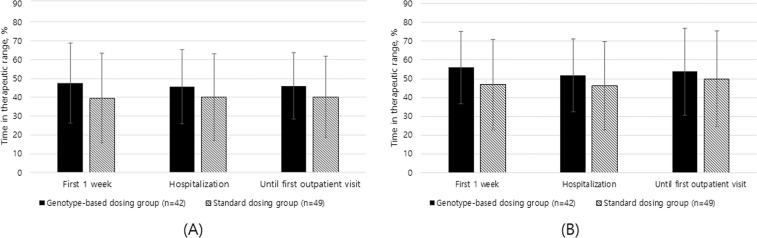
Percentage of time in therapeutic range (1.8–3.2) after heart valve replacement. (**A**) Traditional method (**B**) Rosendaal method. Data were expressed as the mean ± S.D.

The genotype-based dosing group revealed a trend toward higher TTR_Traditional_ and TTR_Rosendaal_, compared with the standard dosing group, however, statistical significance was not achieved (Fig. 1). For the first week following treatment initiation, patients in the genotype-based dosing group had a higher mean TTR_Rosendaal_ than those in the standard dosing group (55.9% vs 46.9%, p = 0.059). The mean differences between the TTR_Traditional_ and TTR_Rosendaal_ for the first week following treatment initiation between the genotype-based dosing group and the standard dosing group were 8.1% and 9.0%, respectively. During hospitalization and until the first outpatient visit, the genotype-based dosing group achieved roughly 5% higher TTR, however, these differences were not statistically significant.

Considering their high frequency, subgroup analyses were performed to compare the results in patients with mitral valve replacement (MVR) compared with aortic valve replacement (AVR) (Table 3). Among patients with AVR, both measures of TTR were significantly higher during the first week following treatment initiation in the genotype-based dosing group when compared with the standard dosing group [ie, 58.5% vs. 38.1% (p = 0.009) for pTTR_Traditional_; 64.0% vs. 44.6% (p = 0.012), for pTTR_Rosendaal_]. In patients with MVR, the genotype-based dosing group also achieved higher pTTR_Traditional_ and pTTR_Rosendaal_ compared with the standard dosing group, however, these differences were not statistically significant. Analyses of potential differences among patients with other valve replacements demonstrated no statistically significant differences.

**Table 3:** Percentage of time in therapeutic range (1.8–3.2) after stratification by valve position.

	Aortic valve			Mitral valve		
	Genotype-based dosing group (n = 16)	Standard dosing group (n = 18)	P-value	Genotype-based dosing group (n = 20)	Standard dosing group (n = 23)	P-value
**Traditional method (%)**						
First 1 week	58.5 ± 10.1	38.1 ± 26.8	0.009	44.6 ± 23.5	39.6 ± 21.9	0.477
Hospitalization	55.8 ± 14.0	41.4 ± 24.5	0.042	41.1 ± 21.6	36.9 ± 21.9	0.527
Until first outpatient visit	52.1 ± 15.2	39.2 ± 20.0	0.047	43.5 ± 19.8	37.1 ± 21.7	0.327
**Rosendaal method (%)**						
First 1 week	64.0 ± 12.3	44.6 ± 27.3	0.012	53.4 ± 22.5	46.9 ± 23.2	0.369
Hospitalization	60.5 ± 14.3	45.4 ± 25.7	0.041	47.9 ± 22.9	44.0 ± 23.4	0.581
Until first outpatient visit	53.3 ± 22.8	46.2 ± 23.7	0.387	53.5 ± 25.4	48.2 ± 27.1	0.513

## Discussion

Numerous studies have compared genotype-based warfarin dosing with standard warfarin dosing in various populations. A meta-analysis published in 2018 included 18 trials of mostly Caucasian patients with heterogeneous indications for warfarin (eg, atrial fibrillation, deep vein thrombosis, pulmonary embolism, valve replacement prosthesis). This report compared overall differences in TTR between genotype-based warfarin dosing and non-genotype-based warfarin dosing groups and concluded that genotype-based dosing offered better safety with less bleeding compared with conventional dosing strategies. Additional subgroup analyses further revealed significant differences in studies comparing fixed dosing with genotype-based dosing, but not in studies comparing clinical dosing with genotype dosing. They also observed statistically significant differences in Chinese patients but not in Caucasian patients in studies comparing genotype-based dosing with non-genotype-based dosing^16^. Other meta-analyses published previously have failed to show benefits of genotype-based dosing when assessed using pTTR^17,18^. These inconsistent results demonstrate that any potential benefit of applying genotype-based dosing may not be conferred to all patients. Because differences exist among different patient groups (eg, environmental factors, genetic factors), the clinical utility of genotype-guided warfarin dosing may be different among diverse clinical backgrounds and/or ethnicities.

Studies comparing genotype-based warfarin dosing and conventional warfarin dosing focusing on Asian patients with valve replacement prosthesis are scarce. A study by Wu *et al*. included Han Chinese patients with rheumatic valve replacement and compared clinical-based dosing with genotype-based dosing. The dosing equations were adopted from their previous study which included sex, age, body surface area, and amiodarone use (clinical dosing) and VKORC1, CYP2C9, CYP4F2, age, weight, and amiodarone use (genotype dosing). Although a statistically significant difference was observed in time to stable dose, significant differences in TTR between two groups were not observed, results which correlate with the results from this study^19^.

The results presented here reveal a statistically significant difference in pTTR between genotype-based warfarin dosing and standard warfarin dosing in patients with aortic valve replacement. The sensitive function of aortic valve suggests the importance of managing complications in patients receiving aortic valve replacement. The aortic valve is located at a critical junction in the circulatory system which opens to allow blood to travel from the left ventricle to the aorta and on to the body. It had been considered a passive structure that opens and closes in response to changes in transvalvular pressure, however, a recent study suggested that the aortic valve performs greatly refined functions owing to its unique microscopic structure. These functions allow it to adapt to its hemodynamic and mechanical environment^20^. Although much progress has been made in surgical procedures and prosthetic valve design, complications after aortic valve replacement largely affect patient mortality; the reported 30-day and 1-year mortality rates are 2.1% and 4.9%, respectively^21^. Complications after aortic valve replacement include paravalvular aortic regurgitation, dehiscence, stroke, infective endocarditis, aortic dissection, and hemolysis. Stroke was reported at a rate of 7–17% and hemolysis of 5–15%^22^. Left side valve (mitral and aortic) thrombus can cause systemic embolic events such as stroke and myocardial infarction, which adds significance to appropriate management of anticoagulation. Patients early in the postoperative period are particularly vulnerable and require close attention. Therefore, patients with aortic valve replacement may benefit greatly from genotype-based dosing in the initial period of warfarin therapy.

The limitation of our study needs to be addressed. As we focused on the impact of genotype-dosing at the initiation of warfarin therapy, the long-term impact on TTR could not be measured. The sample size may have been small and this could have lowered the power of the study as reflected in the small difference in pTTR between groups. Thus, further larger studies with longer period are required to confirm these results. Studies to allow for a direct comparison of the results from our study as relating to homogeneity of patient population, comparison group, and factors included in dosing calculation were not identified. However, a thorough review of previously reported studies which were designed to compare genotype-based warfarin dosing with conventional dosing, revealed that genotype-based dosing may benefit certain patient groups.

To our knowledge, this is the first study to investigate the effects of genotype-based warfarin dosing in Korean patients with prosthetic valve replacement surgery. Although our study failed to achieve a significant difference of TTR in genotype-guided dosing vs. standard doing, a possible benefit in patients early after aortic valve replacement has been suggested.

## Supplementary information

Checklist.

CRIS Registration.

Study Protocol.

Raw data.
