# Supplementary material for: Genotype-guided warfarin dosing may benefit patients with mechanical aortic valve replacements: randomized controlled study
Source: Sci Rep. 2020 Apr 24;10:6988. doi: 10.1038/s41598-020-63985-7 (PMC7181853; doi:10.1038/s41598-020-63985-7)
Supplement: Supplementary file 2 — CRIS Registration. [file 41598_2020_63985_MOESM2_ESM.docx]

**
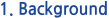
**

[Edit](javascript:goMod('../write/step1.jsp?seq=15576','5'))

| **CRIS Registration Number** | KCT0004586 |
| --- | --- |
| **Unique Protocol ID** | IRB 4-2012-0612 |
| **Public/Brief Title** | Genotype-guided warfarin dosing study |
| **Scientific Title** | Comparative study on standard and pharmacogenetic-guided initial dosing of warfarin in patients with prosthetic heart valves |
| **Acronym** |  |
| **MFDS Regulated Study** | No |
| **IND/IDE Protocol** | No |
| **Registered at Other Registry** | No |
| **Healthcare Benefit Approval Status** | Not applicable |

**
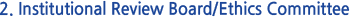
**

[Edit](javascript:goMod('../write/step2.jsp?seq=15576','5'))

| **Board Approval Status** | Submitted approval |
| --- | --- |
| **Board Approval Number** | IRB 4-2012-0612 |
| **Approval Date** | 2012-10-12 |
| **Approval File** | [세브란스IRB-승인.pdf](https://cris.nih.go.kr/cris/file_download.jsp?seq=15576&rf=I&fname=%EC%84%B8%EB%B8%8C%EB%9E%80%EC%8A%A4IRB-%EC%8A%B9%EC%9D%B8.pdf) |
| **Institutional Review Board** |  |
| Name | Severance Hospital, Yonsei University Health System Institutional Review Board |
| Address | 50, Yonsei-ro, Seodaemun-gu, Seoul |
| Telephone | 02-2123-5143 |
| **Data Monitoring Committee** | No |

**
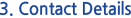
**

[Edit](javascript:goMod('../write/step3.jsp?seq=15576','5'))

| **Contact Person for Principal Investigator / Scientific Queries** |  |
| --- | --- |
| Name | Hye Sun Gwak |
| Title | Professor |
| Email | hsgwak@ewha.ac.kr |
| Telephone | +82-2-3277-4376 |
| Cellular Phone | 01055182898 [confirmed] |
| Affiliation | Ewha Womans University |
| Address | 52, Ewhayeodae-gil, Seodaemun-gu, Seoul |
| **Contact Person for Public Queries** | |
| Name | Kyung Eun Lee |
| Title | associate professor |
| Email | kaylee@cbnu.ac.kr |
| Telephone | +82-2-3277-3052 |
| Cellular Phone | 01089692517 [confirmed] |
| Affiliation | Chungbuk National University |
| Address | 1, Chungdae-ro, Seowon-gu, Cheongju-si, Chungcheongbuk-do |
| **Contact Person for Updating Information** | |
| Name | Kyung Eun Lee |
| Title | associate professor |
| Email | kaylee@cbnu.ac.kr |
| Telephone | +82-2-3277-3052 |
| Cellular Phone | 01089692517 [confirmed] |
| Affiliation | Chungbuk National University |
| Address | 1, Chungdae-ro, Seowon-gu, Cheongju-si, Chungcheongbuk-do |

**
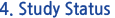
**

[Edit](javascript:goMod('../write/step4.jsp?seq=15576','5'))

| **Study Site** | Single |
| --- | --- |
| **Overall Recruitment Status** | Completed |
| **Date of First Enrollment** | 2013-01-21 , Actual |
| **Target Number of Participant** | 200 |
| **Primary Completion Date** |  |
| **Study Completion Date** |  |
| **Recruitment Status by Participating Study Site 1** | |
| Name of Study Site | Yonsei University Health System, Severance Hospital |
| Recruitment Status | Completed |
| Date of First Enrollment | 2013-01-21 , Actual |

**
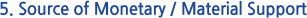
**

[Edit](javascript:goMod('../write/step5.jsp?seq=15576','5'))

| **Source of Monetary/Material Support 1** |  |
| --- | --- |
| Organization Name | Ewha Womans University |
| Organization Type | University |
| Project ID |  |

**
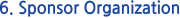
**

[Edit](javascript:goMod('../write/step5.jsp?seq=15576','5'))

| **Sponsor Organization 1** |  |
| --- | --- |
| Organization Name | Ewha Womans University |
| Organization Type | University |

**
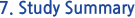
**

[Edit](javascript:goMod('../write/step7.jsp?seq=15576','5'))

| **Lay Summary** | Purpose: To investigate whether initial doses according to VKORC1, CYP2C9, and CYP4F2 genotypes are safer and more effective than conventional empirical doses in patients undergoing prosthetic valve replacement surgery and taking warfarin for the first time. Background and hypothesis: Warfarin is a drug that has a narrow therapeutic range and shows individual differences in dose-response, and is influenced by various factors of patients such as interaction with various drugs, diet, and disease state. The anticoagulant effect can change, which is why the drug needs special monitoring. INR is ideal for being within the therapeutic range, but there are many factors that make it out of the goal. Pharmacogenomics of warfarin is being actively investigated around the world, and an organization called the International Warfarin Pharmacogenomics Consortium (IWPC) has been created to study this. To date, research on warfarin and genotyping has been published in the United States, Japan, and other countries, and recently, studies on Koreans are beginning to be published. We have identified genetic and non-genetic factors that influence the maintenance of patients undergoing heart valve replacement and have established a dose-prediction prediction system based on these factors. Therefore, based on this, this study aims to confirm the warfarin dose according to genotype. Clinical study plan: The subjects of the study were those who underwent cardiac valve replacement and taking warfarin who meet the following criteria and do not meet the exclusion criteria. Selection criteria: Patients who require more than 3 months of warfarin after surgery for heart valve replacement; Exclusion Criteria: 1) Minors under 20 years old, 2) Patients with serious liver disease, kidney disease, 3) Patients with cancer and patients with risk of bleeding such as gastric ulcer. In this study, a blood sample was collected from a patient and a genetic test was performed to determine the dose of warfarin and present the dose to the patient. * Evaluation criteria, evaluation methods, and interpretation methods (statistical analysis methods) In comparing the two groups, the time taken to reach the target INR (2.0-3.0) was constructed by using the Kaplan-Meier method to construct a survival curve and comparing the time-to-group reach by the log-rank test. Analysis of the number of patients reaching the target INR and the occurrence of adverse events (INR≥4, vitamin K use, bleeding, thrombogenesis, myocardial infarction, stroke, death) on days 1-7, 2, 6, and 3 months Use Chi-square. 95% confidence interval is considered to be significant when P <0.05. The statistics program uses SPSS 12.0K for WINDOWS. |
| --- | --- |

**
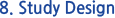
**

[Edit](javascript:goMod('../write/step8.jsp?seq=15576','5'))

| **Study Type** | | Interventional Study |
| --- | --- | --- |
| **Study Purpose** | | Treatment |
| **Phase** | | Not applicable |
| **Intervention Model** | | Parallel |
| **Blinding/Masking** | | Single |
| **Blinded Subject** | | Subject |
| **Allocation** | | RCT |
| **Intervention Type** | | Drug |
| **Intervention Description** | | In this study, after receiving informed consent from the patient, 2.5 mg was administered to the standard dose group (age 65 or older or less than 50 kg) after being assigned to two groups of genotype and standard dose groups using a random table. Alternatively, an initial dose of 5 mg is administered and blood samples are taken at an outpatient visit before surgery, and a genetic test is performed therefrom. |
| **Number of Arms** | | 2 |
| **Arm 1** | Arm Label | Genotype-based dosing group |
|  | Target Number of Participant | 100 |
|  | Arm Type | Experimental |
|  | Arm Description | In this study, after receiving informed consent from the patient, 2.5 mg was administered to the standard dose group (age 65 or older or less than 50 kg) after being assigned to two groups of genotype and standard dose groups using a random table. Alternatively, a prospective study in which an initial dose of 5 mg is administered and a blood sample is taken at an outpatient visit before surgery, a genetic test is performed from this, and a randomly assigned dose is administered. The model yielded the following equation: estimated initial warfarin dose (mg) = 11.305 - 2.082 x (number of VKORC1 rs9934438 T allele) - 1.615 x (number of CYP2C9 rs1057910 C allele) - 0.037 x age (year) + 0.983 x (CYP4F2 rs2108622 AA=1, GA or GG = 0). |
| **Arm 2** | Arm Label | Standard dosing group |
|  | Target Number of Participant | 100 |
|  | Arm Type | No intervention |
|  | Arm Description | The standard dosing group is given an initial dose of 2.5 mg (if older than 65 years of age or weighs less than 50 kg) or 5 mg in the same manner as conventional treatment. |

**
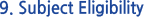
**

[Edit](javascript:goMod('../write/step9.jsp?seq=15576','5'))

| **Condition(s) / Problem(s)** | | * Diseases of the circulatory system  Cardiac Valve Prosthesis |
| --- | --- | --- |
| **Rare Disease** | | No |
| **Inclusion Criteria** | Gender | Both |
|  | Age | 19 Year ~ No Limit |
|  | Description | Patients requiring more than 3 months of warfarin after prosthetic valve replacement |
| **Exclusion Criteria** | | 1) Minors under 20 years old <br />2) patients with severe liver disease or kidney disease <br />3) Patients with cancer and patients with risk of bleeding such as gastric ulcer |
| **Healthy Volunteers** | | No |

**
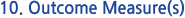
**

[Edit](javascript:goMod('../write/step10.jsp?seq=15576','5'))

| **Type of Primary Outcome** | /Safety/Efficacy |
| --- | --- |
| **Primary Outcome(s) 1** | |
| Outcome | International Normalized Ratio |
| Timepoint | after warfarin start at day 1-7, week 2, 6, and three month later |
| **Secondary Outcome(s) 1** | |
| Outcome | Time until therapeutic INR |
| Timepoint | within study period |
| **Secondary Outcome(s) 2** | |
| Outcome | bleeding complication |
| Timepoint | within study period |

**
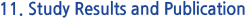
**

[Edit](javascript:goMod('../write/step11.jsp?seq=15576','5'))

| **Result Registered** | Yes Results Upload |
| --- | --- |
| **Final Enrollment Number** | 91 |
| **Number of Publication** | 0 |
| **Results Upload** | [[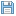](https://cris.nih.go.kr/cris/file_download.jsp?seq=15576&rf=R&fname=RESULTS%20(CRIS).docx) Results Donwload](https://cris.nih.go.kr/cris/file_download.jsp?seq=15576&rf=R&fname=RESULTS%20(CRIS).docx) |
| **Date of Posting Results** | 2019-12-30 |
| **Protocol URL or File Upload** |  |
| **Brief Summary** | This prospective, single-blind, randomized study was designed to evaluate the effect of genotype-based warfarin dosing compared with standard warfarin dosing in Korean patients with mechanical cardiac valves. Patients were assigned to either the genotype-based dosing group or the standard dosing group using stratified block randomization. The genotype-based dosing equation was adopted from a previous study which included VKORC1 rs9934438, CYP2C9 rs1057910, CYP4F2 rs2108622, and age. Primary outcomes included the percentage of time in the therapeutic range (pTTR): i) during the first week following initiation of warfarin therapy, ii) during hospitalization and iii) until the first outpatient visit. A total of 91 patients were included in the analysis, 42 treated with genotype-based warfarin dosing and 49 treated with standard warfarin dosing. The genotype frequency differences of the three SNPs included in this study (ie, VKORC1, CYP2C9, CYP4F2), between the genotype-based dosing and standard dosing groups were not different. The genotype-based dosing group trended toward higher pTTR when compared with the standard dosing group, although this difference was not statistically significant. In patients with aortic valve replacement, TTRTraditional and TTRRosendaal were significantly higher in the genotype-based dosing group when compared with the standard dosing group during the first week following treatment initiation [ie, 58.5% vs. 38.1% (p=0.009) and 64.0% vs. 44.6% (p=0.012), respectively]. Based on the results, genotype-based warfarin dosing may benefit patients who receive aortic valve replacement surgery. |
