# Supplementary material for: Genotype-guided warfarin dosing may benefit patients with mechanical aortic valve replacements: randomized controlled study
Source: Sci Rep. 2020 Apr 24;10:6988. doi: 10.1038/s41598-020-63985-7 (PMC7181853; doi:10.1038/s41598-020-63985-7)
Supplement: Supplementary file 3 — Study Protocol. [file 41598_2020_63985_MOESM3_ESM.docx]

1. **Project summary**

Warfarin is a drug that has a narrow therapeutic range and shows individual differences in dose-response, and is influenced by various factors of patients such as interaction with various drugs, diet, and disease state. The anticoagulant effect can change, which is why the drug needs special monitoring. INR is ideal for being within the therapeutic range, but there are many factors that make it out of the goal. This prospective, single-blind, randomized study was designed to evaluate the effect of genotype-based warfarin dosing compared with standard warfarin dosing in Korean patients with mechanical cardiac valves. Patients were assigned to either the genotype-based dosing group or the standard dosing group using stratified block randomization. The genotype-based dosing equation was adopted from a previous study which included *VKORC1* rs9934438, *CYP2C9* rs1057910, *CYP4F2* rs2108622, and age. Primary outcomes included the percentage of time in the therapeutic range (pTTR): i) during the first week following initiation of warfarin therapy, ii) during hospitalization and iii) until the first outpatient visit.

1. **General information**
   1. **Protocol title, protocol identifying number and date**

- Protocol title: Comparative study on standard and pharmacogenetic-guided initial dosing of warfarin in patients with prosthetic heart valves
- Protocol identifying number: KCT0004586
- Date: 12/30/2019
  1. **Name and address of the sponsor/funder**

None.

- 1. **Name and title of the investigator(s) who is (are) responsible for conducting the research, and the address and telephone number(s) of the research site(s), including responsibilities of each**
- Name and title of the investigators: Hye Sun Gwak (Professor), Byung Chul Chang (Professor)
- Address and telephone number of the research site
- Yonsei University Medical Center, 50-1, Yonsei-ro, Seodaemun-gu, Seoul 03722 Republic of Korea (Telephone: +82-2-2123-5143)
- Ewha Womans University, 52, Ewhayeodae-gil, Seodaemun-gu, Seoul 03760 Republic of Korea (Telephone: +82-2-3277-3052)
  1. **Name(s) and address(es) of the clinical laboratory(ies) and other medical and/or technical department(s) and/or institutions involved in the research**

None.

1. **Rationale & background information**

Warfarin is a drug that has a narrow therapeutic range and shows individual differences in dose-response, and is influenced by various factors of patients such as interaction with various drugs, diet, and disease state [1-3]. The anticoagulant effect can change, which is why the drug needs special monitoring [4]. INR is ideal for being within the therapeutic range, but there are many factors that make it out of the goal.

Pharmacogenomics of warfarin is being actively investigated around the world, and an organization called the International Warfarin Pharmacogenomics Consortium (IWPC) has been created to study this [5]. To date, research on warfarin and genotyping has been published in the United States, Japan, and other countries, and recently, studies on Koreans are beginning to be published. We have identified genetic and non-genetic factors that influence the maintenance of patients undergoing heart valve replacement and have established a dose-prediction prediction system based on these factors [6].

1. **References (of literature cited in preceding sections)**

[1] Tiede DJ, *et al.* Modern management of prosthetic valve anticoagulation. Mayo Clin Proc 1998;73:665-80.

[2] Hirsh J. Oral anticoagulants: Mechanism of action, clinical effectiveness, and optimal therapeutic range. Chest 2001;119:8S-21S.

[3] Gage BF, *et al.* Management and dosing of warfarin therapy. Am J Med 2000;109:481-8.

[4] Rose JP, *et al.* Warfarin sensitivity after mechanical heart valve replacement. Pharmacotherapy 1998;18:856-9.

[5] Klein TE, *et al.* Estimation of the warfarin dose with clinical and pharmacogenetic data. N Engl J Med 2009;360:753-64.

[6] Lee KE, *et al.* Effects of CYP4F2 gene polymorphisms on warfarin clearance and sensitivity in Korean patients with mechanical cardiac valves. Ther Drug Monit 2012;34:275-282

1. **Study goals and objectives**

To investigate whether initial doses according to VKORC1, CYP2C9, and CYP4F2 genotypes are safer and more effective than conventional empirical doses in patients undergoing prosthetic valve replacement surgery and taking warfarin for the first time.

1. **Study design**

- Type of study: Interventional study (a prospective, single-blind, randomized study).
- Research population: Those who underwent cardiac valve replacement and taking warfarin.
- Inclusion criteria: Patients who require more than 3 months of warfarin after surgery for heart valve replacement.
- Exclusion criteria:

1) Minors under 20 years old

2) Patients with serious liver or kidney disease

3) Patients with cancer

4) Patients with risk of bleeding such as gastric ulcer.

1. **Methodology**
   1. **Intervention**

In this study, after receiving informed consent from the patient, 5 mg or 2.5 mg (age 65 or older or less than 50 kg) was administered to the standard dose group after being assigned to two groups of genotype and standard dose groups using a random table. Blood samples are taken at an outpatient visit before surgery, and a genetic test is performed therefrom.

- 1. **Procedures**
- Genotyping: Genotyping for *CYP2C9* (rs1057910), *VKORC1* (rs9934438), and *CYP4F2* (rs2108622) was performed using peripheral blood samples collected prior to the heart valve replacement surgery. Genomic DNA was isolated from EDTA-blood samples using the QIAamp DNA Blood Mini Kit (QIAGEN GmbH, Hilden, Germany) according to the manufacturer’s protocol. The TaqMan genotyping assay was conducted using the real time PCR system (ABI 7300, ABI, Forster City, CA, USA) according to the manufacturer’s protocol.
- Initial warfarin dose estimation: The model yielded the following equation: estimated initial warfarin dose (mg) = 11.305 - 2.082 x (number of *VKORC1* rs9934438 T allele) - 1.615 x (number of *CYP2C9* rs1057910 C allele) - 0.037 x age (year) + 0.983 x (*CYP4F2* rs2108622 AA=1, GA or GG = 0).
- International normalized ratio (INR): INR readings were checked on a daily basis during hospitalization and subsequently according to treating physician preferences.
  1. **Measurements**
- International Normalized Ratio (after warfarin start at day 1-7, week 2, 6, and three month later)
- Time until therapeutic INR (within study period)
- bleeding complication (within study period)
  1. **Randomization**

Patients were assigned to either the genotype-based dosing group or the standard dosing group using stratified block randomization. Strata were defined as a combination of sex (male vs. female) and age (≥65 vs. <65 years); the block size was 6 subjects and patients were blinded to their assigned arm.

- 1. **Blinding**

The patient is blind to the intervention.

- 1. **Stopping rules**
- Patients were excluded if they stopped using the drugs or did not adhere to the study protocol.
- The study would be terminated early if there were serious safety issues.
  1. **Graphic outline**


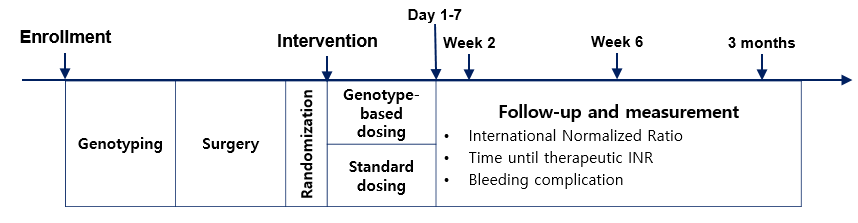


1. **Safety considerations**

The most common and significant adverse events of warfarin is bleeding mainly due to an over-anticoagulation. Bleeding symptoms include gum bleeding, hematuria, bloody stool, nasal bleeding, or hemoptysis. Other adverse events include nausea, vomiting, abdominal pain and loss of appetite. If the symptoms (mentioned above) or all other complaints, which occur during or after the treatment, the administration should be stopped immediately.

1. **Follow-up**

- INR: after warfarin start at day 1-7, week 2, 6, and three month later
- Time until therapeutic INR: within study period
- Bleeding complication: within study period

1. **Data management and statistical analysis**
   1. **Data management**

Appropriate computer programs will be run to verify the accuracy of the database.

- 1. **Statistical analysis**

In comparing the two groups, the time taken to reach the target INR (2.0-3.0) was constructed by using the Kaplan-Meier method to construct a survival curve and comparing the time-to-group reach by the log-rank test. Analysis of the number of patients reaching the target INR and the occurrence of adverse events (INR≥4, vitamin K use, bleeding, thrombogenesis, myocardial infarction, stroke, death) on days 1-7, 2, 6, and 3 months using Chi-squared test. A p-value of < 0.05 was considered statistically significant. All analyses were performed with the IBM SPSS Statistics version 20 Software (International Business Machines Corp., New York, USA).

1. **Quality assurance**

The collected data will be monitored and reviewed every 3 month by the investigators. The chief investigator is responsible for data verification to assess the accuracy, completeness, or representativeness of study data by comparing the study data to original data sources (e.g., medical records).

1. **Expected outcomes of the study**

The benefits of this research extend to potential improvements in the warfarin treatment. Results of this study could be used to develop and implement individually tailored intervention strategy especially for determining the initial dose of warfarin.

1. **Dissemination of results and publication policy**

The chief investigator is Hye Sun Gwak and she had overall responsibility for a study, whereas Principal Investigator for this clinical study is Byung Chul Chang and he had direct clinical responsibility for patients.

1. **Duration of the project**

- IRB approval: Sep 2012 to Dec 2012
- Enrollment: Jan 2013 to Aug 2017
- Follow-up: Jan 2013 to Dec 2017
- Analysis and discussion: Jan 2018 to Dec 2019

1. **Problems anticipated**

- Patients’ reluctance to participate in the study: This could cause slow recruitment of patients. We will monitor screening closely to improve timely recruitment.
- Protocol deviation or violation: This could result in patient exclusion. We will educate researchers prior to and during the recruitment.

1. **Project management**

Kyung Eun Lee (KEL), Byung Chul Chang (BCC) and Hye Sun Gwak (HSG) conceived the initial work and designed the research method. Jeong Yee (JY) and Gwan Yung Lee (GYL) analyzed genotypes. JY, GYL, Jee Eun Chung (JEC) and Jong Mi Seong (JMS) formed the datasets and performed statistical analyses.

1. **Ethics**

The study was reviewed and approved by the Ethics Committee of the Severance Hospital Institutional Review Board and conducted in accordance with the Declaration of Helsinki and Good Clinical Practice guidelines (IRB 4-2012-0612). Informed consent was obtained from all participants prior to the enrollment.

1. **Informed consent forms**
2. **Study title**

Comparative study on standard and pharmacogenetic-guided initial dosing of warfarin in patients with prosthetic heart valves

1. **Backgroud information and objectives of the study**

Large inter-patient variability of response to warfarin has been observed, and this could be explained by genetic polymorphisms. Therefore, this study aimed to contribute to optimal anticoagulation treatment by investigating pharmacogenetic-guided or conventional empirical dose of warfarin in patients undergoing prosthetic valve replacement surgery and taking warfarin for the first time.

1. **Expected outcomes of the study**

Although there have been many studies about the association between warfarin and genetic polymorphism, a few studies have been conducted in Korea. Many studies have been performed the effects of genetic polymorphisms, age, sex, ethnicity, smoking status and co-medication on warfarin dose. However, it is difficult to apply the results from foreign counties to Korea because genetic polymorphisms vary by ethnicity. Therefore, the studies on Korean patients are needed for clinical application. By investigating whether genotype-based doses of warfarin are safer and more effective than conventional empirical doses, it might be possible to implement individually tailored intervention strategy especially for determining the initial dose of warfarin.

1. **Risk of the study and stop rules**

In genotype-based dosing group, you may receive a higher dose than the empirical dose of warfarin and it might result in bleeding or thrombogenesis. If the patients show poor compliance, or are willing to withdraw from the study, the study will be discontinued. The study would be terminated early if there were serious safety issues in the interim analysis (n=60 and/or 100).

1. **Research site, investigator, and total expected number of patients.**
2. Research site: Yonsei University Medical Center and Ewha Womans University
3. Principal investigator: Byung Chul Chang
4. Total expected number of patients: 200
5. Duration of the project: 60 months after IRB approval
6. **Methodology**

This study is to investigate whether genotype-based doses of warfarin are safer and more effective than conventional empirical doses. Patients, who voluntarily agree take part in this study, will be randomly assigned to receive genotype-based or standard dose of warfarin. In genotype-based dosing group, dose is decided by genotype, and after day 3, dose is adjusted by INR in the same way between two groups. For genotyping, we use a portion of the blood already collected for laboratory test under your agreement; therefore, additional visit is not needed. Blood samples will not be used for any other purpose other than this study. All information will be kept confidential.

1. **Information provided**

All information will be kept confidential, and information can be provided on request limited to yours. Information will be provide privately by the study staff in charge.

1. **Sample storing, handling and anonymization**

Collected samples are stored in deep freezer until 5 years after end of the study. Demographic and clinical information are locked with a security program that only the study staff will be able to access.

1. **Confidentiality**

All information will be kept confidential, and information will be collected without patient identifiers by assigning random number to each patient. When the results are published, your identity will not be disclosed.

However, medical files can be viewed by authorized persons as part of monitoring personnel, audit personnel, ethics review committee, and health authorities to inspect the procedures and reliability. By signing this consent form, you permit us to view your medical records.

1. **Voluntary participation/withdrawal**

Participation in this study is voluntary. You may refuse to participate or withdraw from the study at any time and there will be no negative consequences for you.

If you agree to participate in this study, you will receive

- description of this study, and
- copy of signed informed consent

If you have any questions or desire further information about this study, you can contact

- 02) 2228-8481 - Byung Chul Chang
- 02) 3277-3052 - Kyung Eun Lee

If you have any concerns about your rights as a research participant, contact the Research Participant Complaint Line in the Yonsei University Medical Center by phone at 02-2228-0430~4 (Institutional Review Board for human rights) or 02-2228-0450~4 (Human research protection center).

Date ____________________

Principal Investigator: Byung Chul Chang (sign)
